# The Role of Life Stages in the Sensitivity of *Hediste diversicolor* to Nanoplastics: A Case Study with Poly(Methyl)Methacrylate (PMMA)

**DOI:** 10.3390/toxics12050352

**Published:** 2024-05-10

**Authors:** Beatriz Neves, Miguel Oliveira, Carolina Frazão, Mónica Almeida, Ricardo J. B. Pinto, Etelvina Figueira, Adília Pires

**Affiliations:** 1 Department of Biology, University of Aveiro, 3810-193 Aveiro, Portugal; beatrizrneves@ua.pt; 2 Centre for Environmental and Marine Studies, Department of Biology, University of Aveiro, 3810-193 Aveiro, Portugal; migueloliveira@ua.pt (M.O.); carolina.frazao@ua.pt (C.F.); monica.alm@ua.pt (M.A.); efigueira@ua.pt (E.F.); 3 CICECO-Aveiro Institute of Materials, Department of Chemistry, University of Aveiro, 3810-193 Aveiro, Portugal; r.pinto@ua.pt

**Keywords:** PMMA, nanoparticles, polychaetes, benthic invertebrates, biochemical markers, behavior, life stages

## Abstract

The presence of plastic particles in oceans has been recognized as a major environmental concern. The decrease in particle size increases their ability to directly interact with biota, with particles in the nanometer size range (nanoplastics—NPs) displaying a higher ability to penetrate biological membranes, which increases with the decrease in particle size. This study aimed to evaluate the role of life stages in the effects of poly(methyl)methacrylate (PMMA) NPs on the polychaete *Hediste diversicolor*, a key species in the marine food web and nutrient cycle. Thus, behavioral (burrowing activity in clean and spiked sediment) and biochemical endpoints (neurotransmission, energy reserves, antioxidant defenses, and oxidative damage) were assessed in juvenile and adult organisms after 10 days of exposure to spiked sediment (between 0.5 and 128 mg PMMA NPs/Kg sediment). Overall, the results show that *H. diversicolor* is sensitive to the presence of PMMA NPs. In juveniles, exposed organisms took longer to burrow in sediment, with significant differences from the controls being observed at all tested concentrations when the test was performed with clean sediment, whereas in PMMA NP-spiked sediment, effects were only found at the concentrations 8, 32, and 128 mg PMMA NPs/Kg sediment. Adults displayed lower sensitivity, with differences to controls being found, for both sediment types, at 8, 32, and 128 mg PMMA NPs/Kg sediment. In terms of Acetylcholinesterase, used as a marker of effects on neurotransmission, juveniles and adults displayed opposite trends, with exposed juveniles displaying increased activity (suggesting apoptosis), whereas in adults, overall decreased activity was found. Energy-related parameters revealed a generally similar pattern (increase in exposed organisms) and higher sensitivity in juveniles (significant effects even at the lower concentrations). NPs also demonstrated the ability to increase antioxidant defenses (higher in juveniles), with oxidative damage only being found in terms of protein carbonylation (all tested NPs conditions) in juveniles. Overall, the data reveal the potential of PMMA NPs to affect behavior and induce toxic effects in *H. diversicolor*, with greater effects in juveniles.

## 1. Introduction

Since the beginning of the mass production of plastics, in the 1940s and 1950s [1], these materials’ multiple applications promoted their increased production, reaching, in 2022, 400.3 million tons worldwide [2]. A considerable amount of the plastics produced were synthesized with the goal of being a disposable product, frequently poorly managed at end of life. Thus, the amount of plastics that reach the environment has been increasing along with its production, reaching the aquatic environment and ultimately the oceans [3]. Floating plastics have been reported since the 1970s [4], and once they have entered the marine environment, through direct disposal, inland waterways, wastewater outflows, and transport by wind or tides [5], these can migrate through the action of currents, winds, and tides. The physical abrasion of these movements combined with other abiotic (UV radiation, salinity, and natural cycles of temperature and light) and biotic factors like microbial activity leads to degradation, embrittlement, and plastic fragmentation into smaller plastic particles in the millimeter to micrometer size—microplastics (MPs)—and nanometer size—nanoplastics (NPs) [6,7,8,9,10]. The decrease in size allows for an increase in surface area, which means that in the same area, there are many more particles. These smaller particles pose a great threat to marine organisms, since they are more available to be incorporated into living organisms (e.g., through feeding or by penetrating biological membranes) and exert potential pernicious effects [11].

Among the reported effects are decreased energy levels, oxidative stress, DNA damage, apoptosis, inflammation, growth impairment, decreased locomotor activity, malformations, impaired fertility, and mortality [12,13,14,15]. However, the toxicity of these particles has several modulating factors, and the effects of particles may vary according to species and even life stage [12]. Several aspects associated with the particles’ characteristics and the surrounding environment may modulate their properties and thus their effects on biota, hence making the assessment of the particles and their effects in the natural environment difficult [16].

Poly(methyl)methacrylate (PMMA) is an amorphous polymer from the acrylate family [17] discovered in the 1930s and having had its first applications four years later [18]. It is a transparent plastic with high resistance to sunshine exposure, good thermal stability, optical properties, and compatibility with human tissues [19] and is used for biomedical applications, such as bone cement [20]; optical applications, such as contact lenses [21]; solar applications, such as dye-sensitized solar cells (DSSCs) [22]; and nanotechnology, such as plastic chip electrodes (PCEs) [23]. The elevated regularity of its use has made this polymer among the most detected in the environment (e.g., [24,25]). However, despite its wide use, there is a limited number of studies focused on PMMA NPs’ biological effects in marine animals [26,27,28].

Polychaetes are the most abundant and essential group of organisms [29] and are considered good models in toxicity studies [30,31]. *Hediste diversicolor* is an important species in estuarine and coastal food webs, used by aquaculture companies as food [32]. These organisms spend most of their life buried in sediment, where they build Y- and U-shaped galleries, and surface only to feed, being deposit feeders, which is when they are most vulnerable to predation [32,33,34]. Their feeding and burrowing activities contribute to sediment mixing, substrate oxygenation, resuspension, and distribution of nutrients and contaminants [35]. Considering the widespread dispersion of plastics and that NPs tend to sediment, benthic species such as *H. diversicolor* are exposed and are thus potential targets for their potential effects.

Studies have shown that *H. diversicolor* is sensitive to MPs and NPs exposure, even at low concentrations, displaying behavioral, physiological, biochemical, and immunological alterations [28,36,37,38,39,40,41]. Although this species has been used in several ecotoxicity studies and the juvenile stage is considered the most vulnerable life stage [42], there is very little information regarding polychaete juveniles, especially with NPs.

Thus, this study aimed to compare the sensitivity of two life stages of *H. diversicolor*, juvenile and adult, to PMMA NPs in terms of behavior alterations and biochemical endpoints associated with neurotransmission, energy metabolism, and oxidative stress.

## 2. Materials and Methods

### 2.1. Test Organisms

Adult polychaetes caught at a reference site [43] in Ria de Aveiro (40.6331° N, −8.7367° W) were acclimated, for two weeks, to laboratory conditions in artificial salt water (ASW) (salinity 28, pH 8.00) and clean sediment (in a 3:1 ratio), under continuous aeration, in a temperature-controlled room (16 ± 1 °C). The sediment (clean fine sand; organic matter: 0.32%) was collected in a low-contaminated area [43,44], near the organism’s collection site. Once in the laboratory, sediment was sieved with a 1 mm mesh to remove large debris [44]. During that period, polychaetes were fed, every 3 days, with 10 mg of fish food (protein; 46.0%; lipids, 11.0%) per worm, and the water was renewed partially every week [30,45].

The acclimated adult polychaetes were induced to reproduce by increasing the temperature of the corresponding aquaria from 16 °C to 23 °C [46]. Offspring were allowed to grow under laboratory conditions (clean ASW, salinity 28, and pH 8.00) and clean sediment (in a ratio of 3:1) in a temperature-controlled room (16 ± 1 °C) under continuous aeration [46]. Polychaetes were fed twice a week with the same commercial fish food provided to adults (5 mg per worm) [31,47].

Juveniles (3 months) with sizes between 2 and 3 cm and adults (8 months) with sizes between 6 and 8 cm were selected for the experimental assays. Polychaetes were carefully checked for any signs of injury and, in the case of adults, to make sure the animals were not in the reproduction stage.

### 2.2. Nanoplastics Synthesis and Characterization

PMMA NPs were synthesized based on the method described by Manuel et al. (2022) [48]. Briefly, PMMA NPs were prepared by mini-emulsion of methyl methacrylate (MMA), with sodium dodecyl sulphate (SDS) as a stabilizer. The NPs acquired were purified for a week by dialysis (MWCO 6–8 K), with water renewal every 24 h to remove the excess SDS.

The morphological characteristics (size and shape) of the synthesized PMMA particles were analyzed by scanning electron microscopy (SEM) (Hitachi; SU-70, Japan). The hydrodynamic size and the polydispersity index (PDI) of the particles were assessed by dynamic light scattering (DLS) analysis and the zeta potential by electrophoretic light scattering (ELS) (Zetasizer Nano ZS; Malvern, UK). The behavior of the particles (hydrodynamic size and PDI) was studied up to 96 h in ultrapure water and test media (artificial seawater: salinity 28 and pH 8.00).

### 2.3. Experimental Design

Selected adults (90) and juveniles (216) were randomly distributed per six experimental conditions (0, 0.5, 2, 8, 32, and 128 mg PMMA NPs/Kg sediment), with 3 replicates per condition. The NPs concentrations were chosen based on data available on microplastics concentrations in the environment and earlier research on nanoplastics concentrations and marine invertebrates, including polychaetes [13,31,48,49,50,51]. Morgado et al. (2022) also demonstrated that most of the particles found in sediment from estuaries were sized <1000 µm [52], and Shi et al. (2024) detected environmental concentrations of nanoplastics at levels of 0.3–488 µg/L [53]. Exposure was performed through sediment spiking [54]. The concentrations tested were prepared by adding the corresponding amount of nanoplastics stock suspension or Mili-Q water (control) to six separate beakers containing wet sediment. Sediment was thoroughly mixed for ±10 min and was allowed to rest for 2 days. Then, ASW was added carefully not to disturb the sediment.

Experiments were performed in 1 L glass aquaria filled with sediment and artificial seawater (1:2), with continuous aeration, temperature of 16 ± 1 °C, salinity 28, and pH 8.0. Aeration was added, and sediment was allowed to rest for 1 day.

Organisms were exposed for 10 days and were fed every 3 days with commercial fish food, along with partial water changes. After 10 days, burrowing tests were conducted; then, the polychaetes were frozen and stored at −80 °C until biochemical analysis.

### 2.4. Burrowing Assay

Burrowing behavior was evaluated based on the procedure described by Silva et al. (2020) [39] with slight modifications. In this assay, the burrowing behavior of the polychaetes was assessed in clean or in sediment contaminated with the same level of NPs as in the exposure period. This test was performed in both sediment types to assess if polychaetes could distinguish the clean from the contaminated sediments and investigate if they had a preference for one of the sediment types by burrowing faster. Additionally, by testing in contaminated sediment, we can better replicate what could happen in the field. In the test, each polychaete was gently placed in a container (15 cm in diameter and 10 cm in height), filled with 5 cm (height) of test sediment (clean or contaminated sediment) and 1 cm (above the sediment) of clean artificial seawater, and the time the animal took to completely burrow was recorded. A total of 7 adult polychaetes were tested in the control and the other 7 in contaminated sediment, and 18 juvenile polychaetes were tested, 9 in control sediment and the other 9 in contaminated sediment.

### 2.5. Biochemical Analyses

For the biochemical analyses, the organisms were weighed and homogenized in 0.1 M potassium phosphate buffer (pH 7.4) by using an ultrasonic homogenizer (Sonics Vibra-Cell VC130 Ultrasonic Processor, Sonics & Materials, Inc., Newtown, CT 06470 USA). The homogenates were separated into three sets of microtubes: one for the determination of lipid peroxidation and glycogen content; the second to determine cholinesterase and electron transport system activities, where the homogenate was centrifuged at 3300× *g* for 3 min at 4 °C; and the third for post-mitochondrial supernatant (PMS) isolation to determine catalase, superoxide dismutase, and glutathione S-transferases activities and protein carbonylation levels, with the homogenates being centrifuged at 10,000× *g* for 20 min at 4 °C [55].

The protein content from all three fractions was determined by the biuret method by using bovine serum albumin (BSA) standards (0, 2.5, 5, 10, 20, and 40 mg/mL) [56]. The reaction took place at room temperature for 10 min, and absorbance was read at 540 nm with a microplate reader (MOBI UV/Vis Microplate Spectrophotometer, MicroDigital Co., Ltd., Seongnam, Korea).

#### 2.5.1. Neurotransmission

Acetylcholinesterase (AChE) activity was determined based on Elman’s method [57] with adaptations for microplates [58]. The degradation rate of acetylthiocholine was determined by monitoring the increase in the yellow color at 412 nm, due to the binding of the thiocholine with 5,5-dithio-bis (2-nitrobenzoic acid). The results were expressed as nmol/mg of protein (ε = 13,600 M^−1^ cm^−1^).

#### 2.5.2. Energy-Related Parameters

Electron transport system (ETS) activity was measured based on King and Packard (1975) [59] with modifications [60]. Absorbance was read at 490 nm for 5 min in 25 s intervals. The amount of formazan formed was calculated by using ε = 15,900 M^− 1^ cm^−1^, and the results were expressed as nmol/min/mg of protein.

Glycogen (GLY) content was determined by using the phenol–sulfuric acid method according to Dubois et al. (1956) [61]. After the incubation of the sample with phenol and sulfuric acid for a period of 30 min, absorbance was read at 492 nm, and the results were expressed as mg/mg of protein.

#### 2.5.3. Antioxidant Enzyme Response

Superoxide dismutase (SOD) activity was determined according to Beauchamp and Fridovich (1971) [62], i.e., by using the reaction of nitro blue tetrazolium (NBT) with superoxide radicals to form NBT diformazan. After a 20 min incubation period, absorbance was read at 560 nm. SOD activity was expressed in units (U) per mg of protein, where U corresponds to the amount of enzyme that inhibited NBT diformazan formation by 50%.

Catalase (CAT) activity was measured following Oliveira et al. (2010) [63] adapted to a microplate reader, and absorbance was read at 240 nm. The results were calculated by using ε = 43.5 M^−1^ cm^−1^ and expressed in µmol of H_2_ O_2_ consumed/min/mg of protein.

Glutathione S-transferases (GSTs) activity was determined following an adaptation of Habig et al.’s (1974) method [64], in which GSTs catalyze the reaction of the conjugation of a 1-chloro-2,4-dinitrobenzene (CDNB) substrate with the thiol group of GSH (reduced glutathione), forming a thioether (ε = 9.6 mM^−1^ cm^−1^) which can be monitored by the increase in absorbance at 340 nm. The results were expressed as nmol/min/mg of protein.

#### 2.5.4. Oxidative Damage

Protein carbonylation (PC) levels were measured by the quantification of carbonyl groups by using the 2,4-Dinitrophenylhydrazine (DNPH) alkaline method [65] with adaptations [66]. Absorbance was read at 450 nm, and the concentration of carbonyl groups was expressed in nmol/mg of protein, by using 22.308 M^−1^ cm^−1^ as the molar extinction coefficient of the carbonyl-dinitrophenylhydrazine adduct [65].

Lipid peroxidation (LPO) levels were determined by the quantification of thiobarbituric acid reactive substances (TBARSs), which are formed in the reaction between LPO by-products (such as malondialdehyde—MDA) and 2-thiobarbituric acid (TBA). Absorbance was read at 532 nm, and the results were calculated by using the molar extinction coefficient of MDA (ε = 1.56105 M^−1^ cm^−1^) and expressed in nmol/mg of protein [67].

### 2.6. Statistical Analyses

The statistical analysis was made by using IBM SPSS Statistics for Windows, Version 29.0.2.0 (SPSS Inc., Chicago, USA. Before the analyses, the behavioral and biochemical data were checked for normal distribution (Shapiro–Wilk test, *p* > 0.05) and homogeneity of variances (Levene’s test) [68]. For each behavioral and biochemical marker, differences between treatment groups were evaluated by two-way analysis of variance (ANOVA), followed by Tukey’s test, using the interaction between the polychaetes’ life stage and PMMA NPs concentrations as factors. When normality was not met, data were submitted to non-parametric testing such as Kruskal–Wallis one-way analyses on ranks (*p* < 0.05).

## 3. Results

### 3.1. Nanoplastics Synthesis and Characterization

**Figure 1:**
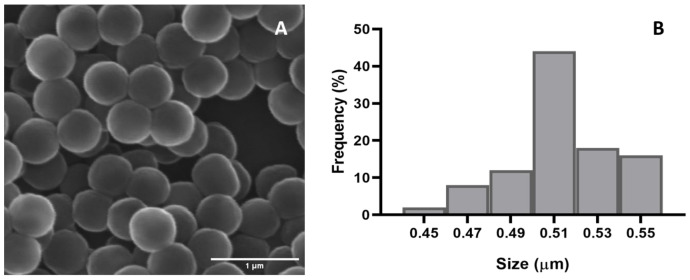
Scanning electron microscopy image of the PMMA particles (**A**) and their corresponding size distribution (**B**).

Scanning electron microscopy showed that the PMMA NPs presented a spherical shape, as presented in Figure 1A, with an average size of 513 nm (Figure 1B). The −12 mv zeta potential revealed that particles exhibited a moderately stable nature. The data from the hydrodynamic size and PDI of the particles from 0 to 96 h are displayed in Table S1. In ultrapure water, at 0 h, the particles displayed a hydrodynamic size of 1678 nm (±138.4 nm) and a PDI of 0.724, whereas in artificial seawater, the particles displayed a hydrodynamic size of 5780 nm (±178.7 nm) and a PDI of 0.934. After 96 h, the particles displayed little variation in hydrodynamic size and PDI (1557 ± 92.40 nm, 0.877) when compared to 0 h, whereas, in artificial seawater, the particles displayed a decrease in hydrodynamic size to 2987 ± 285.6 nm and a PDI of 0.402.

### 3.2. Mortality

No significant differences in mortality were observed under any of the tested conditions. In the assays with adults, a mortality rate of 1.1% was observed at the concentrations 2, 32, and 128 mg PMMA NPs/Kg sediment. In the assays with juveniles, mortality of 0.92% in the control and 0.46% in the concentration 0.5 mg PMMA NPs/Kg sediment were observed.

### 3.3. Behavioral Responses

**Figure 2:**
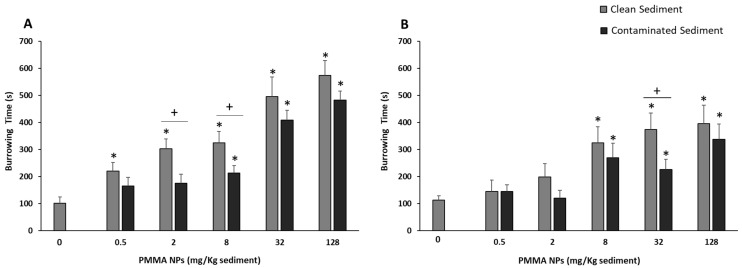
Burrowing time of juveniles (**A**) and adults (**B**) of *Hediste diversicolor* tested in clean and PMMA NPs-spiked sediment after 10 days of exposure to PMMA NPs. The asterisk (*) represents significant differences (*p* < 0.05) from the controls. The plus sign (+) represents significant differences (*p* < 0.05) between sediment types.

The burrowing behavior of juveniles and adults of *H. diversicolor* is shown in Figure 2. PMMA NPs had a significant effect (all tested concentrations) on the time juveniles took to burrow in clean sediment when compared with control organisms. In PMMA NPs-spiked sediment, exposed juveniles only displayed differences to the controls when exposed to 8, 32, and 128 mg PMMA NPs/Kg sediment (*p* < 0.05; Figure 2A). When comparing the burrowing time for the two sediment types, significant differences were found. Polychaetes took longer to burrow when placed in clean sediment, although statistically significant differences were only found for 2 and 8 mg PMMA NPs/Kg sediment.

Adult polychaetes’ behavior displayed overall lower sensitivity to PMMA NPs exposure than that of juveniles. Significant increases in burrowing time when compared with controls were also found for both sediment types but were only significant for 8, 32, and 128 mg PMMA NPs/Kg sediment-exposed organisms (*p* < 0.05; Figure 2B). Comparing the behavior for both sediment types, significant differences were only observed for organisms exposed to 32 mg PMMA NPs/Kg sediment, with animals taking more time to burrow in clean sediment (Figure 2B).

When comparing both life stages, no significant differences between juveniles and adults were found in clean sediment, whereas in contaminated sediment, i.e., 32 and 128 mg PMMA NPs/Kg sediment, juveniles took significantly more time to burrow (*p* < 0.05).

### 3.4. Biochemical Responses

Overall, the basal levels (i.e., observed in controls) were significantly different in adults and juveniles, with the exception of AChE activity.

Nevertheless, all parameters presented significant differences (*p* < 0.05) between the two life stages at all PMMA NPs tested concentrations.

#### 3.4.1. Neurotransmission

**Figure 3:**
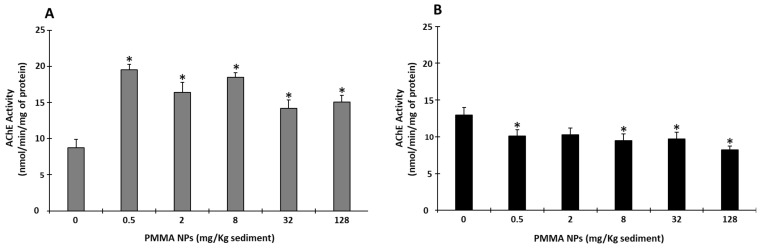
Neurotransmission: acethylcholinesterase (AChE) activity in juvenile (**A**) and adult (**B**) *Hediste diversicolor* after 10 days of exposure to PMMA NPs. Data represented are mean values ± standard errors. The asterisk (*) represents significant differences (*p* < 0.05) from the controls.

AChE activity in juveniles exposed to sediment spiked with PMMA NPs (all tested concentrations) was significantly higher than in juvenile controls (*p* < 0.05; Figure 3A). However, AChE activity in adults displayed an opposite trend, with significantly lower enzyme activity at all PMMA NPs concentrations (*p* < 0.05; Figure 3B), except for 2 mg PMMA NPs/Kg sediment.

#### 3.4.2. Energy-Related Parameters

**Figure 4:**
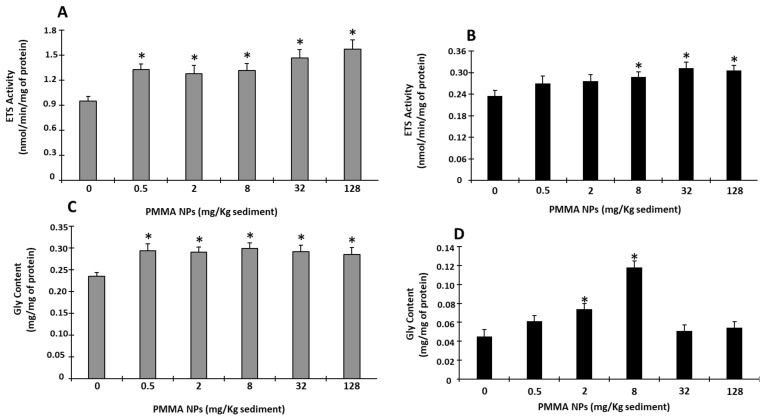
Energy-related parameters: electron transport chain (ETS) (**A**,**B**) and glycogen (GLY) (**C**,**D**) in juvenile (**A**,**C**) and adult (**B**,**D**) *Hediste diversicolor* after 10 days of exposure to PMMA NPs. Data represented are mean values ± standard errors. The asterisk (*) represents significant differences (*p* < 0.05) from the controls.

ETS activity in juveniles was significantly increased after exposure to PMMA NPs sediment (all tested concentrations) (*p* < 0.05; Figure 4A). In adults, ETS activity also showed an increasing tendency, although significant differences from the controls were only found in 8, 32, and 128 mg PMMA NPs/Kg sediment-exposed organisms (*p* < 0.05; Figure 4B).

GLY content in juveniles displayed an increasing trend in PMMA NPs-exposed organisms (all concentrations), with levels significantly higher than in the controls at all PMMA NPs concentrations (*p* < 0.05; Figure 4C). However, in adults, GLY content was only significantly higher than in the controls for 2 and 8 mg PMMA NPs/Kg sediment (*p* < 0.05; Figure 4D).

#### 3.4.3. Antioxidant Enzyme Responses

**Figure 5:**
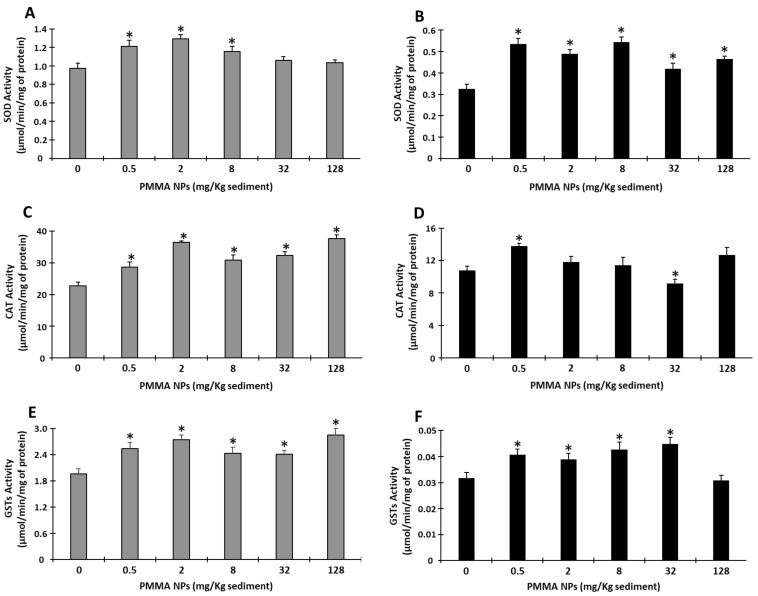
Antioxidant and biotransformation enzymes response: superoxide dismutase (SOD) (**A**,**B**), catalase (CAT) (**C**,**D**), and glutathione-S-transferases (GSTs) (**E**,**F**) measured in juvenile (**A**,**C**,**E**) and adult (**B**,**D**,**F**) *Hediste diversicolor* after 10 days of exposure to PMMA NPs. Data represented are mean values ± standard errors. The asterisk (*) represents significant differences (*p* < 0.05) with the controls.

In juveniles, SOD activity was significantly higher than in the controls at 0.5, 2, and 8 mg PMMA NPs/Kg sediment concentrations, returning to control levels at higher concentrations (*p* < 0.05; Figure 5A). In adults, SOD activity was more sensitive to PMMA NPs exposure, displaying higher values than the controls at all tested PMMA NPS concentrations (*p* < 0.05; Figure 5B).

CAT activity presented an overall increasing tendency with significant differences from the controls found at all tested PMMA NPs concentrations (*p* < 0.05; Figure 5C). Regarding adults, significant increases in CAT activity were found only for 0.5 mg PMMA NPs/Kg sediment, with significant decreases observed for 32 mg PMMA NPs/Kg sediment (*p* < 0.05; Figure 5D).

Glutathione S-transferase (GST) activity in juveniles was significantly higher than in control organisms at all tested PMMA NPs concentrations (*p* < 0.05; Figure 5E). In adults, GST activity was also significantly higher than in the controls at all the tested PMMA NPs concentrations, except the highest (128 mg PMMA NPs/Kg sediment) (*p* < 0.05; Figure 5F).

#### 3.4.4. Oxidative Damage

**Figure 6:**
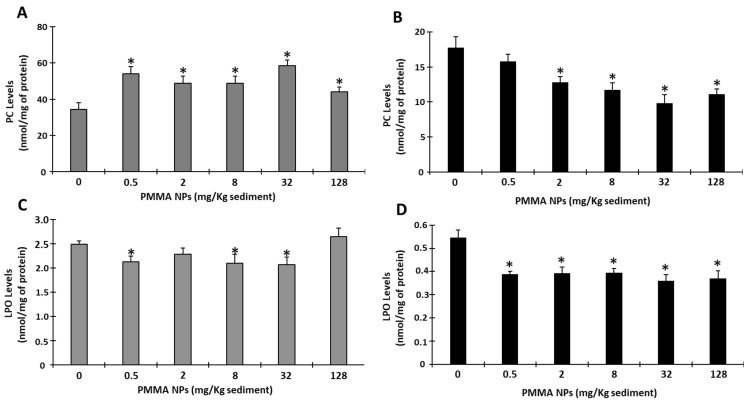
Oxidative damage: protein carbonylation (PC) (**A**,**B**) and lipid peroxidation (LPO) (**C**,**D**) measured in juvenile (**A**,**C**) and adult (**B**,**D**) *Hediste diversicolor* after 10 days of exposure to PMMA NPs. Data represented are mean values ± standard errors. The asterisk (*) represents significant differences (*p* < 0.05) from the controls.

The juveniles’ PC levels were significantly higher than those of the controls at all tested PMMA NPs concentrations (*p* < 0.05; Figure 6A). In adults, there was a decreasing trend, with significant differences to the controls, found at all tested concentrations, except 0.5 mg PMMA NPs/Kg sediment (*p* < 0.05; Figure 6B).

LPO levels in juveniles showed a significant decrease, when compared with the controls, at the concentrations 0.5, 8, and 32 mg PMMA NPs/Kg sediment (*p* < 0.05; Figure 6C), whereas in adults, LPO levels were significantly lower than in the controls at the tested PMMA NPs concentrations (*p* < 0.05; Figure 6C).

## 4. Discussion

To date, there has been a limited number of studies addressing the effects of PMMA NPs on marine organisms, and even fewer studies have considered the effects of spiked sediment. In this study, the effect of PMMA NPs-spiked sediment on *H. diversicolor*, considered ecosystem engineers, was assessed.

After analyzing the PMMA NPs in seawater, the results demonstrate that these particles tend to aggregate, with the particle size increasing in the water column. Although particle size distribution at the lowest concentrations was not characterized, a decrease in aggregation is expected due to the reduced number of particles in sediment [39]. Additionally, the availability of these smaller particles to benthic organisms may be enhanced compared with higher-concentration treatments, since nanoplastics (1 nm–1000 nm) can penetrate cell membranes and cause greater injuries [69]. Several studies reported that NPs can aggregate when present in seawater (artificial and natural) [70]. Furthermore, studies state that in the natural environment, NPs are expected not only to aggregate forming agglomerates but also to interact with other particles and compounds present in water or sediment, increasing NPs density and size [71,72]. Studies have shown that the agglomeration process, along with other factors, can influence the particles’ stability, demonstrating that environmental factors such as organic matter, light exposure, and ions can contribute to both the aggregation and the stability of the particles [73,74,75,76].

An *H. diversicolor* burrowing impairment could provide information on the potential increased susceptibility of these organisms (e.g., inability to avoid predators), as well as on potential effects on the community [77], since a potential impact or total impairment of burrowing/feeding activity affects nutrient resuspension and the oxygenation of sediment. In this study, no mobility issues were observed during burrowing assays, with organisms moving in circular motions along the edge of the container; therefore, the inability to move cannot be the reason for their altered burrowing behavior. Thus, the increase in time required to burrow in sediment does not appear to be associated with an inability to move. The burrowing results obtained in the present study show an overall increase in burrowing both in clean and contaminated sediment, for both life stages. Both juveniles and adults took longer to burrow in clean sediment than the ones tested in contaminated sediment. The fact that organisms take longer to burrow, even in clean sediment, suggests that the delay in burrowing is not associated with avoidance behavior, which would be supported by higher burrowing time in spiked sediment than in the control. More studies are needed to understand the mechanisms involved. Some studies performed similar behavioral tests. For instance, a study evaluating the responses to waterborne polystyrene (PS) NPs of adult *H. diversicolor*, conducted by Silva et al. (2020) [39], showed an increase in burrowing time with the increase in concentration (0.005, 0.05, 0.5 mg of PS NPS/L), apart from the highest two concentrations (5 and 50 mg of PS NPs/L), where the observed reduced burrowing activity, compared to the lowest concentrations, was associated with potential aggregation and lower availability of the particles. Other studies with copper and Graphene Oxide (GO) nanosheets also portrayed the loss of burrowing capacity of organisms [78,79]. AChE is an enzyme responsible, both in vertebrates and invertebrates, for the hydrolysis of the neurotransmitter acetylcholine in cholinergic synapses, which mainly regulates neuromuscular transmission. This enzyme’s reduced activity is associated with muscle impairment, compromising the organism’s behavior [26,80]. This endpoint, used as a neurotoxicity biomarker, could help explain if the increase in burrowing time is associated with a decreased ability to move, swim, or burrow [80,81]. In adult polychaetes, the decreased activity tendency portrayed, along with the increase in burrowing time, could suggest that AChE activity inhibition was responsible for the impairment in burrowing behavior. However, in juveniles, an opposite trend was observed, with AChE presenting higher activity levels than in the controls. Previous studies have also reported increased AChE activity in *H. diversicolor* after exposure to contaminants like copper [82], which was associated with the possible rapid synthesis of AChE enzyme, since its activity was measured after the burrowing tests, that were performed 48 h after the end of exposure to the contaminant [82]. However, as the burrowing assay and AChE activity in the present study were performed immediately after exposure, it is unlikely that recovery could occur and alter AChE activity. Another possible explanation for the AChE activity increase appointed in the literature is cell apoptosis, a form of programmed cell death with important roles in development, tissue homeostasis, and various diseases [83]. Studies have been investigating the relation between AChE and apoptosis and have demonstrated that AChE also plays an important role as a regulator of apoptosis (caused by oxidative stress) and that apoptogenic stimuli may increase AChE levels in organisms, including invertebrates [84]. Moreover, at higher concentrations, higher NPs aggregation may have occurred, which could have prevented the action of PMMA NPs at higher concentrations, since larger particles cannot penetrate cell membranes; therefore, organisms exposed to lower concentrations were more affected.

Overall, except for AChE activity, the biochemical endpoints’ basal levels were higher in juveniles than in adults, which may be associated with a higher metabolic need associated with ontogenic development, which endows these organisms with a higher ability to face stressors [85].

Overall, in the present study, the juvenile stage appeared to be more affected by exposure to PMMA NPs-spiked sediment. Energy metabolism has an essential role in the adaptation and tolerance of organisms to stressful situations [86]. Previous studies showed that aquatic invertebrates exposed to contaminants can increase energy expenditure, considered a cell protection mechanism [87]. In this study, the levels of glycogen, a stored energy source, increased in juveniles at all PMMA NPs concentrations and, in adults, at the concentrations 2 and 8 mg PMMA NPs/Kg sediment. Other studies found no significant alterations in the energy content of polychaetes. Van Cauwenberghe et al. 2015 [88] did not observe alterations in sugar, carbohydrate, and lipid content in the polychaete *Arenicola marina* exposed to polystyrene microspheres (10, 30, and 90 μm), and total energy content was not altered in *H. diversicolor* exposed to GO nanoflakes [89]. On the other hand, *H. diversicolor* exposed to PS NPs GO nanosheets for 28 days displayed decreased sugar content, which suggests that polychaetes use an immediately available energy source to fuel their defense mechanisms against GO nanosheet toxicity, since stored energy, as lipids and glycogen, was preserved [78].

Electron transport system activity (ETS) has been used to determine metabolic capacity in response to environmental stressors, like contaminants [87]. In this study, ETS activity was significantly higher than in the control at all PMMA NPs exposure concentrations in juveniles and at the three highest concentrations in adults. This ETS activity increase suggests an allocation of energy to activate defenses, such as antioxidant enzymes, and prevent damage [86]. Previous studies that exposed polychaetes to PS NPs [39] and GO nanoparticles [78,90] also reported an increase in ETS activity.

SOD activity is considered the first line of defense against xenobiotics, converting superoxide ions into hydrogen peroxide. In the present study, an increase in this enzyme’s activity was observed, in juveniles and adults, which suggests that the organisms were able to initiate a defense against ROS (reactive oxygen species) induced by PMMA NPs. The ability of NPs to induce SOD activity in *H. diversicolor* has been previously reported [39]. Yu et al. (2018) also observed that juvenile *Eriocheir sinensis* crabs exposed to 500 nm PS-NPs also displayed increased SOD activity in the hepatopancreas [91].

CAT, which catalyzes the transformation of hydrogen peroxide into water and oxygen, was also increased in juveniles, following the trend of SOD activity. However, in adults, the response pattern of this enzyme was not so clear, increasing its activity in organisms exposed to 0.5 mg PMMA NPs/Kg sediment and decreasing its activity in 32 mg PMMA NPs/Kg sediment-exposed organisms. This decreased activity response suggests a potential toxic effect on the organisms exposed to 32 mg PMMA NPs/Kg sediment. A lower ability to remove hydrogen peroxide may have consequences for the cells and lead to peroxidative damage, inflammation, and apoptosis [92]. An increase in the antioxidant enzymes SOD and CAT was also observed in the visceral mass, mantle, and gill of *Corbicula fluminea* exposed to 80 nm PS-NPs [93].

The activity of GSTs, in addition to their role in phase II biotransformation, also has an antioxidant role in protecting the cells from damage from toxic compounds, such as xenobiotics and their metabolites, by catalyzing their conjugation with reduced glutathione. In the present study, GST activity in juveniles increased in organisms exposed to PMMA NPs, suggesting metabolic alterations and activation of detoxifying mechanisms. Adult polychaetes also demonstrated increased GST activity, signaling the activation of detoxifying efforts. However, the organisms exposed to the highest concentration showed no significant differences from the controls, suggesting either the activation of other mechanisms of defense or the possible aggregation of the PMMA NPs at higher concentrations, reducing the availability of these nanoparticles to benthic organisms. Although particle size distribution at the lowest concentrations was not characterized, a decrease in aggregation is expected due to the reduced number of particles in sediment [39]. Additionally, the availability of these smaller particles to benthic organisms may be enhanced compared to higher-concentration treatments, since nanoplastics can penetrate cell membranes and cause greater injuries.

The assessment of damage endpoints allows a better understanding of the ability of the organisms to deal with NPs exposure. In the present work, no PC and the oxidative modification resulting from the oxidation of the side chains of proteins were observed in adults. PC is one of the most harmful and irreversible oxidative protein modifications. Carbonyl stress is related to biomolecule malfunction, immunogenicity, inflammation, cell toxicity, and apoptosis [94,95]. Under stressful conditions, such as exposure to contaminants, PC levels are expected to increase, as demonstrated in studies with marine organisms, including polychaetes. PC levels were significantly lower than in the controls in 2, 8, 32, and 128 mg PMMA NPs/Kg sediment-exposed organisms. These results suggest that *H. diversicolor* efficiently activated defense mechanisms that prevented this type of damage to proteins. Pires et al. (2022) also reported a decrease in PC levels when adult *H. diversicolor* were exposed to GO nanoflakes at low concentrations, suggesting that *H. diversicolor* effectively counteracted the effect of the contaminant by activating antioxidant defenses [78]. However, the present study revealed that juveniles showed high sensitivity to protein damage, with a general significant increase in PC levels in exposed organisms indicating that SOD, CAT, and GST activities, although increased, could not prevent oxidative damage. These endpoints have been shown to be sensitive in *H. diversicolor* exposed to arsenic [77] and to the combination of arsenic and PS NPs [96], *Ficopomatus enigmaticus* exposed to carboxylated carbon nanotubes [97], and *Diopatra neapolitana* worms exposed to trace elements (arsenic, zinc, iron, manganese, lead, and tin) [98]. LPO, a parameter associated with greater production of free radicals, which can lead to an increase in the permeability of the cell membranes, rupture, and possible cell death, was, in general, lower than in the controls. The obtained data suggest that in both juveniles and adults, the activation of defense/repair mechanisms decreased this type of damage after PMMA NPs exposure. This result is in agreement with Silva et al. (2023), who reported a decrease in LPO levels in *H. diversicolor* exposed to 50 nm PMMA NPs and suggested efficient neutralization of ROS by the activation of the antioxidant defense system [28]. Similar results were also found for MPs. Suitable neutralization of ROS was also previously hypothesized in mussel *Mytillus* spp. exposed to PS MPs (2–6 μm, 32 µg/L, 7 days of exposure) [99].

## 5. Conclusions

Plastics remain constant contaminants in the marine environment, and the results of their breakage have been shown to affect this ecosystem and its species. With NPs sinking and accumulating in sediments, benthic species become a potential target. This study aimed to assess the effects of PMMA NPs on marine invertebrates, more specifically, *H. diversicolor*, and its impact on this species but also the role of the life stages in these effects.

In order to obtain a more realistic approach, tests were performed through sediment spiking, as it is expected to be an important route of exposure often neglected in laboratory studies. Overall, the data show that the presence of NPs may compromise polychaetes, particularly in earlier life stages. The findings show that in terms of behavior and oxidative stress, the juvenile stage, in general, is the most affected life stage, since juveniles need more time to burrow, have higher cellular damage, and have higher antioxidant enzyme activity. The decreased burrowing capacity can lead to less sediment mixing, which translates into less nutrient resuspension, affecting the involved biota, which also become more vulnerable to predators, which could affect the future of *H. diversicolor* populations. Furthermore, oxidative damage was observed in juveniles, with an increase in protein carbonylation, showing that despite the activation of some defenses by the increase in SOD, CAT, and GST activities, they were inefficient against PMMA NPs’ effects. Adults, however, did not portray oxidative damage, showing that defense mechanisms were able to counter PMMA NPs.

The obtained results showed that the life stage can drastically alter organisms’ responses to PMMA NPs. As organisms evolve in their life cycle, their internal processes change accordingly, and those differences in their metabolism can affect their response to stress, either increasing resistance or vulnerability.

Since juvenile and larval stages are the most important life stages for the maintenance of populations, it is imperative that these are also considered when testing the toxicity of any contaminant.

## Data Availability

The data presented in this study are available on request from the corresponding author.

## Supplementary Materials

The following supporting information can be downloaded at: https://www.mdpi.com/article/10.3390/toxics12050352/s1, Table S1: Characterization of PMMA particles in terms of hydrodynamic size and polydispersity at different time points. Data are presented as mean ± standard deviations.
