# Supplementary material for: The Role of Life Stages in the Sensitivity of Hediste diversicolor to Nanoplastics: A Case Study with Poly(Methyl)Methacrylate (PMMA)
Source: Toxics. 2024 May 10;12(5):352. doi: 10.3390/toxics12050352 (PMC11126148; doi:10.3390/toxics12050352)
Supplement: Supplementary file 1 [file toxics-12-00352-s001.zip › toxics-2963363-supplementary.pdf]

Supporting Information for

The Role of Life Stages in the Sensitivity of *Hediste diversicolor* to Nanoplastics: A Case Study with Poly(Methyl)Methacrylate (PMMA)

Beatriz Neves [1], Miguel Oliveira [2], Carolina Frazão [2], Mónica Almeida [2], Ricardo J. B. Pinto [3], Etelvina Figueira [2] and Adília Pires [2,*]

[1] Department of Biology, University of Aveiro, 3810-193 Aveiro, Portugal
[2] Centre for Environmental and Marine Studies, Department of Biology, University of Aveiro, 3810-193 Aveiro, Portugal
[3] CICECO-Aveiro Institute of Materials, Department of Chemistry, University of Aveiro, 3810-193 Aveiro, Portugal
* Correspondence: adilia@ua.pt

PMMA particles		0h	24h	48h	72h	96h
**Ultrapure Water**	Hydrodynamic size (nm)	1678 ± 138.4	2542 ± 172.0	928.4 ± 25.68	1848 ± 170.0	1557 ± 92.40
	Polydispersity Index	0.724	0.911	0.569	0.835	0.877
**Artificial Sea water**	Hydrodynamic size (nm)	5780 ± 178.7	5574 ± 646.0	5030 ± 129.5	5968 ± 926.7	2987 ± 285.6
	Polydisperse Index	0.934	0.959	0.682	0.797	0.402

Table S1 – Characterization of PMMA particles in terms of hydrodynamic size and polydispersity at different time points. Data are presented as mean ± standard deviation.
